# Genetic analysis of the *ATP11B* gene in Chinese Han population with cerebral small vessel disease

**DOI:** 10.1186/s12864-022-09051-0

**Published:** 2022-12-12

**Authors:** Wen-Kai Yu, Yun-Chao Wang, Yuan Gao, Chang-He Shi, Yu Fan, Lu-Lu Yu, Zi-Chen Zhao, Shan-Shan Li, Yu-Ming Xu, Yu-Sheng Li

**Affiliations:** 1 Department of Neurology, The First Affiliated Hospital of Zhengzhou University, Zhengzhou University, Zhengzhou, Henan China; 2 National Health Commission Key Laboratory of Prevention and Treatment of Cerebrovascular Diseases, Zhengzhou, Henan China

**Keywords:** Cerebral small vessel disease, ATP11B gene, Variant, Chinese Han population

## Abstract

**Background:**

A loss-of-function mutation in ATPase phospholipid transporting 11-B (putative) (*ATP11B*) gene causing cerebral small vessel disease (SVD) in vivo, and a single intronic nucleotide polymorphism in *ATP11B*: rs148771930 that was associated with white matter hyperintensities burden in European patients with SVD, was recently identified. Our results suggest that *ATP11B* may not play an essential role in SVD in the Chinese population.

**Results:**

We performed target region sequencing including *ATP11B* gene in 182 patients with sporadic SVD, and identified five rare variants and two novel variants of *ATP11B*. A case–control study was then performed in 524 patients and matched 550 controls to investigate the relationship between *ATP11B* and sporadic SVD in the Chinese Han population. Although none of these variants were significantly associated with SVD in our samples, it is important to mention that we identified a novel variant, p. G238W, which was predicted to be pathogenic in silico. This variant was present in our cohort of patients with an extremely low frequency and was absent in the controls.

**Conclusion:**

Our results suggest that *ATP11B* may not play an essential role in SVD in the Chinese population.

**Supplementary Information:**

The online version contains supplementary material available at 10.1186/s12864-022-09051-0.

## Background

Cerebral small vessel disease (SVD) is a syndrome characterized by clinical, neuroimaging, and neuropathological manifestations caused by disorders that affect small cerebral vessels, including arteries, arterioles, capillaries, and venules in the brain [1]. It contributes to approximately 20% of stroke cases and 45% of vascular dementia worldwide [2, 3]. White matter hyperintensities (WMH) is an important neuroimaging marker for diagnosing SVD because of its mild clinical symptoms, especially in the early stages of the disease [1]. Meanwhile, WMH is considered to be mainly caused by breakdown of the blood–brain barrier (BBB), loss of oligodendrocytes, and demyelination [4]. Although there is no exact molecular mechanism of pathogenesis, increasing evidence shows that BBB leakage is associated with SVD [5–7]. Endothelial cells (ECs) are an essential constituent of the BBB, and endothelial dysfunction is assumed to be a critical contributor to SVD [8, 9].

Recently, Rikesh et al. identified a mutation that leads to a truncated protein in the ATPase phospholipid transporting 11-B (putative) (*ATP11B*) gene causing EC dysfunction in a rat model of SVD. They also found an intronic single nucleotide polymorphism (SNP) in *ATP11B*: rs148771930, which was associated with WMH burden in European patients with SVD. These results suggest that loss-of-function mutations in *ATP11B* may result in the WMH of SVD, and rare variants in the *ATP11B* cloud are risk factors for SVD in European population [10]. However, there is no genetic evidence for *ATP11B* mutations in the Chinese population.

Here, we performed a comprehensive *ATP11B* variant screening in sporadic SVD patients and healthy controls to further investigate the relationship between *ATP11B* and sporadic SVD in the Chinese Han population.

## Results

This study enrolled 1074 Chinese Han subjects including 524 patients with SVD and 550 healthy controls. There was no significant statistical difference in age (p = 0.290), sex (p = 0.125) and hypertension (p = 0.108) between SVD patients and control subjects (Supplemental Table 1).

**Fig. 1:**
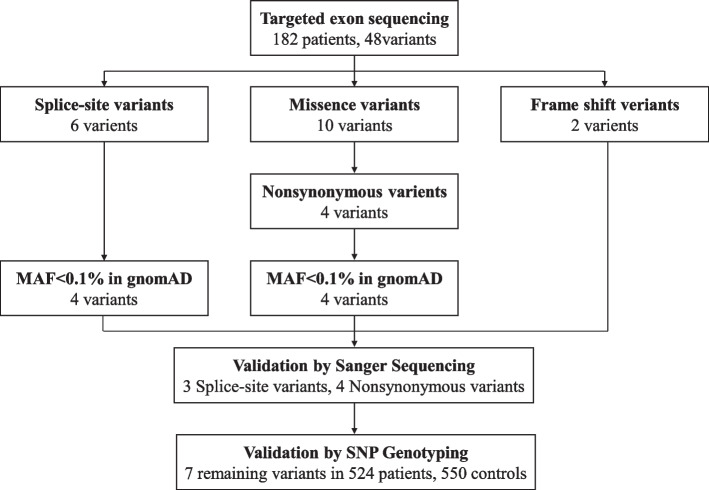
The process of variant filtration

A total of 48 genetic variants were identified after NGS in 182 SVD patients (Supplemental Table 2), and we eventually determined seven variants with potential contribution to disease after variant filtration sanger sequencing validation (Fig. 1).

**Fig. 2:**
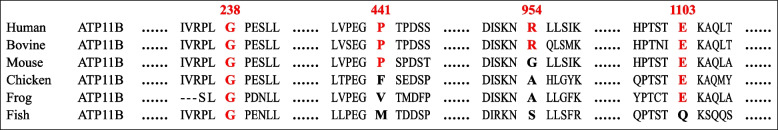
Evolutionary conservation analysis of altered amino acid. red: this amino acid residue is evolutionarily conserve

All seven variants identified in *ATP11B* were heterozygous except p.E1103K. One of the three loci of p.E1103k was homozygous in one patient, and the others were heterozygous carried by two patients. None of these variants appeared to have significantly different allele frequencies between patients and controls (Tables 1 and 2). The altered amino acid influenced by p.G238W is evolutionarily conserved from human to fish (Fig. 2).

**Table 1:** Variants found in *ATP11B* in SVD patients and healthy controls

Position at chr3	SNP	cDNA	Functional change	minor allele frequency		SVD vs Controls	
				SVD (524)	Controls (550)	OR (95% CI)	P
182563253	NA	c.712G > T	p. G238W	0.095%	0	NA	0.305
182583365	rs186142123	c.1322C > T	p. P441L	0.095%	0	NA	0.305
182587011	NA	c.1763-5 T > -	NA	0.095%	0	NA	0.305
182607215	rs143776237	c.2861G > A	p. R954H	0.859%	0.273%	3.167(0.855–11.732)	0.069
182615205	rs201924008	c.3152 + 11A > G	NA	0.286%	0.091%	3.155(0.328–30.379)	0.294
182616549	rs138771155	c.3307G > A	p. E1103K	0.382%	0.091%	4.211(0.470–37.735)	0.162
182631645	rs773539019	c.3319-4G > T	NA	0.191%	0	NA	0.147

Abbreviations: SNP, single nucleotide polymorphism; cDNA, complementary DNA; SVD, small vessel disease; OR, odds ratio; CI, confidence interval; NA, Not available

**Table 2:** Geographical and ethnic differences in minor allele frequency of *ATP11B* variants & protein function prediction of coding variants

Variants in *ATP11B*	SNP ID	minor allele frequency			SIFT Score ^a^	POLYPHEN V2 Score ^b^	Mutation Taster Score ^c^
		This study	gnomAD-Genomes East Asian	gnomAD-Genomes European			
c.712G > T: p. G238W	NA	0.047%	NA	NA	0.001 (D)	0.471 (P)	1 (D)
c.1322C > T: p. P441L	rs186142123	0.047%	0.060%	0	0.101 (T)	0 (B)	0.995 (D)
c.1763-5 T > -	NA	0.047%	NA	NA	NA	NA	NA
c.2861G > A: p. R954H	rs143776237	0.559%	0.260%	0.005%	0.29 (T)	0.002 (B)	0.92 (N)
c.3152 + 11A > G	rs201924008	0.186%	0	0.005%	NA	NA	NA
c.3307G > A: p. E1103K	rs138771155	0.233%	0.390%	0	0.005 (D)	0.221 (B)	1 (D)
c. 3319-4G > T	rs773539019	0.093%	0.190%	0	NA	NA	NA
c.770-1236C > T	rs148771930	NA	0	0.292%	NA	NA	NA

Abbreviations: SNP, single nucleotide polymorphism; gnomAD, Genome Aggregation Database; SIFT, Sorting Intolerant From Tolerant; POLYPHEN, Polymorphism Phenotyping; NA, Not available

^a^Sorting Intolerant From Tolerant Score Pred: D = damaging T = tolerated

^b^Polymorphism Phenotyping Score Pred: B = benign P = possibly damaging D = probably damaging

^c^Mutation Taster Score Pred: A = disease causing automatic D = disease causing N = polymorphism P = polymorphism automatic

## Discussion

The *ATP11B* gene encodes probable phospholipid-transporting ATPase IF protein (NM_014616.3). ATP11B is a catalytic component of the P4-ATPase flippase complex that catalyzes the hydrolysis of ATP coupled to the transport of aminophospholipids from the outer to the inner leaflet of various membranes [11–13]. Rikesh et al. hypothesized that loss of ATP11B may cause endothelial dysfunction because of blocked vesicular transport from the trans-Golgi network to the plasma membrane. They showed that HSP90α, which is secreted from dysfunctional ECs, is an important factor in interrupting the maturation of oligodendrocytes, and eventually contributes to impaired myelination. Therefore, *ATP11B* may be closely associated with the WMH of SVD [10].

In our study, we failed to identify the SNP: rs148771930, which was previously reported to be associated with WMH burden in European patients with SVD; this might be due to the fact that different ethnic groups have different genetic backgrounds (Table 2). However, we identified five rare variants and two novel variants by NGS of *ATP11B* in Chinese Han patients with sporadic SVD and healthy controls. Among them, p.G238W, p.P441L (rs186142123), c.1763-5 T > -, and c.3319-4G > T (rs773539019) were carried by the patients only. None of these variants were significantly associated with SVD in the Chinese samples. Given that the sample size was too small to capture these low-frequency variants, larger samples are needed to clarify a more comprehensive distribution of *ATP11B* in various populations and areas.

It is worth mentioning that we identified a novel variant, p.G238W. It is located in exon 9 of *ATP11B*, a region encoding a part of probable phospholipid-transporting ATPase IF, and p. G238W seems to have pathogenic prediction in silico based on three different bioinformatics analyses (Table 2). In addition, the altered amino acid influenced by p.G238W is evolutionarily conserved across multiple species (Fig. 2). This evidence, together with the extremely low minor allele frequency of this rare variant in our study (Table 2), suggest that the potential effect on the biologic al function of ATP11B merits further investigation.

## Conclusions

In conclusion, our study failed to reproduce the association between the polymorphism of *ATP11B* and SVD in the Chinese Han population. Further studies in various populations are needed to investigate the role of *ATP11B* in the pathogenesis of SVD.

## Methods

### Subjects

This study recruited 524 Chinese Han sporadic SVD patients from the Neurology Department of the First Affiliated Hospital of Zhengzhou University between March 2018 and October 2021. Diagnosis was performed by both neurologists and neuroimaging [14], and the severity of WMH was quantified on fluid-attenuated inversion recovery (FLAIR) and T2-weighted magnetic resonance imaging scans (MRI). The following inclusion and exclusion criteria were used:

The inclusion criteria were as follows: (1) age > 18 years; (2) the Fazekas score of WMH is from 2 to 3 on T2-FLAIR brain MRI scans [15]; (3) cognitive dysfunction & memory impairment caused by vascular dementia; (4) psychiatric disorder influenced by cerebrovascular disease.

Exclusion criteria: (1) a cerebral infarct larger than one third the volume of the cerebellar hemisphere identified on diffusion-weighted imaging; (2) dementia due to confirmed neurodegenerative diseases; (3) an aneurysm (diameter > 3 mm), or a history of cerebral vascular malformation or aneurysmal subarachnoid hemorrhage; (4) mental diseases diagnosed according to the diagnostic and statistical manual of mental disorders-5; (5) intracranial infection, traumatic brain injury, brain tumors, and epilepsy; (6) a family history of cerebrovascular disease or vascular dementia, or harboring mutations in *NOTCH3*, *HTRA1*, and *COLA1/2* genes [16].

In addition, 550 ethnicity-, age-, and sex-matched healthy individuals were enrolled as controls. They were confirmed to be healthy by on history of neurological and psychiatric diseases, normal neuroimaging and laboratory examination. In addition, hypertension and advanced age are well-known risk factors for SVD [17, 18]; therefore, we performed Student’s t-tests and Chi-square tests to assess the differences in these potential confounders between patients and controls.

### Next generation sequencing (NGS) and bioinformatics analysis

In our previous work, we performed target region sequencing including the *ATP11B* gene in 182 patients with sporadic SVD [19]. Genomic DNA was extracted from the peripheral blood collected from patients and controls using a genomic DNA extraction kit (#DP1101, BioTeke, Beijing) based on standard protocols. NGS of whole exons and adjacent intron regions of *ATP11B* (Supplemental Table 3) was carried out on an Illumina MiSeq high-throughput sequencing platform (Illumina, San Diego, CA, USA) provided by Phylotree (Zhengzhou) Biotechnology Co., Ltd.

The raw sequencing data was converted into a collection of small fragment sequences called Reads, in the file format Fastaq. Sequencing reads were aligned to human reference genome 19 using the Burrows-Wheeler Aligner (BWA) [20]. Single-nucleotide variant (SNV) calling was performed using both Genome Analysis Toolkit (GATK) [21] and Varscan programs [22], and the called SNV data were then combined. The Annovar program was used for SNV annotation [23]. The functional effect of non-synonymous variants was assessed by the PolyPhen-2, SIFT, and Mutation Taster [24–26]. The evolutionary conservation analysis of altered amino acids was performed by aligning the sequences of *ATP11B* across multiple species.

### Variant filtration and validation by sanger sequencing

Variant filtration was performed after bioinformatics analysis of raw sequencing data from NGS. First, variants in intron areas (except for splice-site variants), 3 ' UTR and 5 ' UTR areas, and synonymous variants were ruled out. We then queried the Genome Aggregation Database (gnomAD) (http://gnomad.broadinstitute.org) and filtered out variants whose minor allele frequency (MAF) was greater than 0.1% based on gnomAD. Finally, sanger sequencing was performed to confirm the remaining variants. Specific primers for exons 9, 13, 17, 25, 27, 28 and 29 of *ATP11B* were designed using Primer 6 software (Supplemental Table4). These exons and corresponding exon–intron boundaries were amplified using the polymerase chain reaction (PCR). DNASTAR Lasergene MegAlign (v7.1.0) and Chromas (v2.33) were used to conduct sequence alignment. All variants not validated by sanger sequencing were exclude (Fig. 1).

### SNP genotyping

SNaPshot analysis of SNP genotypes was performed to confirm the remaining variants after variant filtration in the samples of patients and controls, using a SNaPshot Multiplex kit system (Applied Biosystems, Foster City, CA, USA). Specific primers were designed using the Primer 3 Software (Supplemental Table 5). SNaPshot results were analyzed using Gene Mapper v4.1 software (Foster City, CA, USA).

### Statistical analysis

Pearson’s ${x}^{2}$ test was used to compare allele frequencies between patients and controls. The Student’s t-test was used to assess the difference in age between patients with SVD and health controls (age at SVD onset for patients vs. age at recruitment for healthy controls). The chi-square test was used to assess the difference in sex and history of hypertension between the two groups. The p value, OR, and 95% confidence intervals were calculated using SPSS (v.21.0), and statistical significance was set at p < 0.05.

## Supplementary Information

**Additional file1.**

## Data Availability

All data generated or analysed during this study are included in this article and it’s supplementary material. The target capture sequencing data including *ATP11B* can be freely and openly accessed at the Sequence Read Archive of the National Center for Biotechnology Information (NCBI) (https://www.ncbi.nlm.nih.gov/sra) database with the ID SUB11084355. These data have been processed and will be released on January 1, 2023. *ATP11B* gene sequences of multiple species were obtained from Uniport (https://www.uniprot.org/).

## Abbreviations

ATP11B: ATPase phospholipid transporting 11-B
SVD: Small vessel disease
WMH: White matter hyperintensities
BBB: Blood-brain barrier
ECs: Endothelial cells
SNP: Single nucleotide polymorphism
FLAIR: Fluid-attenuated inversion recovery
MRI: Magnetic resonance imaging scans
NGS: Next Generation Sequencing
BWA: Burrows-Wheeler Aligner
SNV: Single-nucleotide variant
GATK: Genome Analysis Toolkit
PCR: Polymerase chain reaction
gonmAD: Genome Aggregation Database
MAF: Minor allele frequency
