# Supplementary material for: Genetic analysis of the ATP11B gene in Chinese Han population with cerebral small vessel disease
Source: BMC Genomics. 2022 Dec 12;23:822. doi: 10.1186/s12864-022-09051-0 (PMC9746074; doi:10.1186/s12864-022-09051-0)
Supplement: Supplementary file 1 — Additional file1. [file 12864_2022_9051_MOESM1_ESM.docx]

**Supplemental Table 1. Demographic and clinical characteristics of the study subjects.**

| Variables | Patients with SVD  (n=524) | Heathy controls  (n=550) | *P* value |
| --- | --- | --- | --- |
| Age, years; mean±SD | 63.96±8.47 (Onset age) | 63.42±8.31 (Recruitment age) | 0.290 |
| Gender, *n* (%) |  |  | 0.125 |
| Male | 312 (59.5%) | 302 (54.9%) |  |
| Female | 212 (40.5%) | 248 (45.1%) |  |
| Hypertension, *n* (%) |  |  | 0.108 |
| positive | 356 (67.9%) | 348 (63.3%) |  |
| negative | 168 (32.1%) | 202 (36.7%) |  |

**Supplementary Table 2:** **Detailed information of the 48 *ATP11B* (chr3, NM_014616) variants identified in NGS.**

| Position | Ref Allele | Alt Allele | Gene Region | Predicted Protein Variants |
| --- | --- | --- | --- | --- |
| 182511351 | C | T | UTR5 | c.-197C>T |
| 182545931 | A | C | exonic | exon3:c.168A>C:p.P56P |
| 182546017 | C | A | intronic | NA |
| 182547406 | G | A | intronic | NA |
| 182553749-182553749 | T | - | intronic | NA |
| 182554830 | A | G | intronic | NA |
| 182563253 | G | T | exonic | exon9:c.712G>T:p.G238W |
| 182575631 | G | T | intronic | NA |
| 182576923 | T | A | intronic | NA |
| 182577198 | G | A | intronic | NA |
| 182577248-182577248 | A | - | intronic | NA |
| 182577260 | C | T | intronic | NA |
| 182583365 | C | T | exonic | exon13:c.1322C>T:p.P441L |
| 182584172 | G | A | exonic | exon14:c.1560G>A:p.S520S |
| 182584205 | A | G | exonic | exon14:c.1593A>G:p.E531E |
| 182587011-182587011 | T | - | splicing | exon17:c.1763-5T>- |
| 182587126 | G | A | splicing | exon17:c.1866+7G>A |
| 182590350 | A | G | intronic | NA |
| 182590354 | C | T | intronic | NA |
| 182591561 | A | T | intronic | NA |
| 182597314 | G | A | exonic | exon20:c.2283G>A:p.G761G |
| 182598831 | G | A | intronic | NA |
| 182598838 | A | G | intronic | NA |
| 182598851 | A | G | intronic | NA |
| 182598863 | A | G | intronic | NA |
| 182603693 | G | A | intronic | NA |
| 182603881 | G | A | intronic | NA |
| 182605442 | T | C | exonic | exon24:c.2784T>C:p.Y928Y |
| 182607215 | G | A | exonic | exon25:c.2861G>A:p.R954H |
| 182614493 | T | C | exonic | exon26:c.2988T>C:p.F996F |
| 182615205 | A | G | splicing | exon27:c.3152+11A>G |
| 182616549 | G | A | exonic | exon28:c.3307G>A:p.E1103K |
| 182631645 | G | T | splicing | exon29:c.3319-4G>T |
| 182631766-182631766 | C | - | exonic | exon29:c.3436delC:p.P1146fs |
| 182631770-182631770 | C | - | exonic | exon29:c.3440delC:p.T1147fs |
| 182631789-182631789 | - | T | splicing | exon29:c.3452+7->T |
| 182631792 | C | A | splicing | exon29:c.3452+10C>A |
| 182636128 | T | G | UTR3 | c.*236T>G |
| 182636129 | C | G | UTR3 | c.*237C>G |
| 182636294 | T | C | UTR3 | c.*402T>C |
| 182636751 | A | C | UTR3 | c.*859A>C |
| 182637172-182637173 | AT | - | UTR3 | c.*1280_*1281delAT |
| 182637840 | C | T | UTR3 | c.*1948C>T |
| 182638524 | G | C | UTR3 | c.*2632G>C |
| 182639109-182639114 | TAAAAG | - | UTR3 | c.*3217_*3222delTAAAAG |
| 182639121 | A | G | UTR3 | c.*3229A>G |
| 182639335 | A | G | UTR3 | c.*3443A>G |
| 182639420-182639421 | AA | - | UTR3 | c.*3528_*3529delAA |

**Supplemental Table 3. The target regions for NGS of *ATP11B* gene.**

| **Chr** | **Start position** | **End position** | **Length** | **Gene** | **mRNA** | **Region** |
| --- | --- | --- | --- | --- | --- | --- |
| 3 | 182793002 | 182793501 | 499 | *ATP11B* | NM_014616 | Promoter |
| 3 | 182793502 | 182793758 | 256 | *ATP11B* | NM_014616 | 5'UTR |
| 3 | 182793759 | 182793786 | 27 | *ATP11B* | NM_014616 | exon1 |
| 3 | 182820259 | 182820376 | 117 | *ATP11B* | NM_014616 | exon2 |
| 3 | 182828119 | 182828209 | 90 | *ATP11B* | NM_014616 | exon3 |
| 3 | 182829671 | 182829752 | 81 | *ATP11B* | NM_014616 | exon4 |
| 3 | 182836034 | 182836142 | 108 | *ATP11B* | NM_014616 | exon5 |
| 3 | 182836341 | 182836470 | 129 | *ATP11B* | NM_014616 | exon6 |
| 3 | 182837070 | 182837174 | 104 | *ATP11B* | NM_014616 | exon7 |
| 3 | 182842074 | 182842122 | 48 | *ATP11B* | NM_014616 | exon8 |
| 3 | 182845457 | 182845522 | 65 | *ATP11B* | NM_014616 | exon9 |
| 3 | 182848475 | 182848557 | 82 | *ATP11B* | NM_014616 | exon10 |
| 3 | 182857877 | 182858028 | 151 | *ATP11B* | NM_014616 | exon11 |
| 3 | 182859161 | 182859359 | 198 | *ATP11B* | NM_014616 | exon12 |
| 3 | 182865455 | 182865698 | 243 | *ATP11B* | NM_014616 | exon13 |
| 3 | 182866267 | 182866443 | 176 | *ATP11B* | NM_014616 | exon14 |
| 3 | 182867375 | 182867444 | 69 | *ATP11B* | NM_014616 | exon15 |
| 3 | 182869077 | 182869151 | 74 | *ATP11B* | NM_014616 | exon16 |
| 3 | 182869227 | 182869331 | 104 | *ATP11B* | NM_014616 | exon17 |
| 3 | 182872355 | 182872537 | 182 | *ATP11B* | NM_014616 | exon18 |
| 3 | 182873811 | 182874015 | 204 | *ATP11B* | NM_014616 | exon19 |
| 3 | 182879495 | 182879649 | 154 | *ATP11B* | NM_014616 | exon20 |
| 3 | 182880878 | 182880981 | 103 | *ATP11B* | NM_014616 | exon21 |
| 3 | 182884752 | 182884898 | 146 | *ATP11B* | NM_014616 | exon22 |
| 3 | 182885950 | 182886010 | 60 | *ATP11B* | NM_014616 | exon23 |
| 3 | 182887585 | 182887713 | 128 | *ATP11B* | NM_014616 | exon24 |
| 3 | 182889409 | 182889548 | 139 | *ATP11B* | NM_014616 | exon25 |
| 3 | 182896699 | 182896765 | 66 | *ATP11B* | NM_014616 | exon26 |
| 3 | 182897302 | 182897406 | 104 | *ATP11B* | NM_014616 | exon27 |
| 3 | 182898606 | 182898772 | 166 | *ATP11B* | NM_014616 | exon28 |
| 3 | 182913860 | 182913994 | 134 | *ATP11B* | NM_014616 | exon29 |
| 3 | 182918022 | 182918104 | 82 | *ATP11B* | NM_014616 | exon30 |
| 3 | 182918105 | 182921633 | 3528 | *ATP11B* | NM_014616 | 3'UTR |
| 3 | 182921634 | 182922133 | 499 | *ATP11B* | NM_014616 | 3'Flanking |

**Supplemental Table 4. Primer sequences used for Sanger sequencing**

| Region of *ATP11B* | Primer sequences |
| --- | --- |
| Exon 9 | F: 5’-TTTAGCAGAGACGGGGTT-3’  R: 5’-CAGGGATCATCAACAAAACA-3’ |
| Exon 13 | F: 5’-TAAGAATCTCAGCATCCATA-3’  R: 5’-TTCCTGCAACTCATCAACTA-3’ |
| Exon 17 | F: 5’-TTCTTGTTCTCAATTAGCCAT-3’  R: 5’-CCCCGACTGTAGAGTAGGTTTG-3’ |
| Exon 25 | F: 5’-ATGTTTGAATGTAGTATGTGA-3’  R: 5’-AAAACTGTCTAGTAACTTGAG-3’ |
| Exon 27 | F: 5’-GTATAGTGCAAGACAGCGTA-3’  R: 5’-CACAGAAAAAAACAGGGA-3’ |
| Exon 28 | F: 5’-TCTTGCTTTACTTGGTCC-3’  R: 5’-GAAACAGGAATGGCTAAT-3’ |
| Exon 29 | F: 5’-GCCTTTCATATGTTTCCCA-3’  R: 5’-AAAAAGCCCTTACCCCAAA-3’ |

**Supplemental Table 5.** **Primer sequences used for SNaPshot analysis**

| Position at chr3 or SNP | Primer sequences |
| --- | --- |
| 182563253 | F: 5’-GGCCCCTGGGCAGCTTTATAGT-3’  R: 5’-ATCTGGCTCCACGAAGCAGGAG-3’ |
| 182587011 | F: 5’-CACCTTCAGGTAACCAATCTAAACTTTCA-3’  R: 5’-AACACACAGTGTAACACAACTGCAGAA-3’ |
| rs138771155 | F: 5’-CAGCTCCTGTCAAGTGGTTCTGCT-3’  R: 5’-GAATGGCTAATTTCCTATTTTCTGACATAATC-3’ |
| rs143776237 | F: 5’-TCGCACCCAGTCTTCCTATTTCTTT-3’  R: 5’-GAAGCCCAGGATGGTCCAATAAAG-3’ |
| rs186142123 | F: 5’-GAAAATGAGATGCAGTTTCGGGAAT-3’  R: 5’-GGACTGGTTCTGAAAGAGGAACTGG-3’ |
| rs201924008 | F: 5’-GGACTTGGATCAACCATCTCGTTACC-3’  R: 5’-TGGGGTTTAAATCTTGGCTTTGGTA-3’ |
| rs773539019 | F: 5’-TGTTCTAATGCTTTTCAGAAGTCCATATC-3’  R: 5’-GTTCATCCTGTTCCAGATAGGATACTCA-3’ |
